# Tentorium Cerebelli: Muscles, Ligaments, and Dura Mater, Part 1

**DOI:** 10.7759/cureus.5601

**Published:** 2019-09-09

**Authors:** Bruno Bordoni, Marta Simonelli, Maria Marcella Lagana

**Affiliations:** 1 Cardiology, Foundation Don Carlo Gnocchi, Milan, ITA; 2 Osteopathy, French-Italian School of Osteopathy, Pisa, ITA; 3 Radiology, IRCCS Fondazione Don Carlo Gnocchi Onlus, Milan, ITA

**Keywords:** tentorium cerebelli, fascia, dura mater, fascial system, cranio, skull

## Abstract

The tentorium cerebelli is an integral part of the reciprocal tension membranes that divide some brain areas: the falx cerebri, the falx cerebelli, and the diaphragma sellae. The article is divided into two parts. The first part reviews the anatomy of the tentorium cerebelli, the dura mater, and the ligaments and cervical muscles connected to the tentorium. The tentorial area may be subject to trauma or surgery and knowledge of anatomy and existing relationships is essential to better understand the clinical picture. The second part reviews the systemic relationships of the tentorium cerebelli. The neurological anatomical information, which links the tentorium to the central and peripheral nervous systems, venous brain drainage. The tentorium is not just a body segment, but a systemic communication tool.

## Introduction and background

The term meninges, from the Greek “membranes”, consist of three layers, which are the dura, the arachnoid, and the pia mater. The embryology of the meningeal layers is very complex and not always clear [1]. The arachnoid and pia mater are derived from the perimedullary mesenchyme (the primitive meninx that consists of mesenchymal cells surrounding the neural tube), therefore, they have an ectodermal origin including the neural crest cells [1]. The dura mater, which originates later, has a mesodermal origin, a part of which would contribute to the formation of the arachnoidal layer; therefore, the latter would then have two embryological origins. The dura mater has the same embryological origin than the fascial system, and we can strongly suggest that this cranial structure is very malleable and able to receive the osteopathic treatment. It contains several fibroblasts, which allow the dura mater to be a flexible structure [1]. Our research group, Foundation of Osteopathic Research and Clinical Endorsement (FORCE), defines the fascia system as follows: “The fascia is any tissue that contains features capable of responding to mechanical stimuli. The fascial continuum is the result of the evolution of the perfect synergy among different tissues, liquids and solids, capable of supporting, dividing, penetrating, feeding, and connecting all the districts of the body, from the epidermis to the bone, involving all the functions and organic structures. The continuum constantly transmits and receives mechanometabolic information that can influence the shape and function of the entire body. These afferent/efferent impulses come from the fascia and the tissues that are not considered as part of the fascia in a biunivocal mode. In this definition, these tissues included epidermis, dermis, fat, blood, lymph, blood and lymphatic vessels, tissue covering the nervous filaments (endoneurium, perineurium, epineurium), voluntary striated muscle fibers and the tissue covering and permeating it (epimysium, perimysium, endomysium), ligaments, tendons, aponeurosis, cartilage, bones, meninges, tongue” [2]. The tentorium cerebelli is a relevant fascial element, able to transmit tensions of cervical and head movement towards the central nervous system and vice versa towards the cervical tract [3-4]. It is a crossroad of mechanical and neurological information. The tentorium suits cranial pressures, changing its shape and curvature, as in idiopathic intracranial hypertension [5]. Its morphology is not a fixed structure but can change according to the mechanical stimuli present [6]. The nervus tentorii has different pathways within the two sides of the skull [7]. This article reviews the neurological anatomical information, which links the tentorium to the central and peripheral nervous systems, as well as the myofascial to the cervical spine and venous brain drainage.

## Review

Cranial dura mater

**Figure 1:**
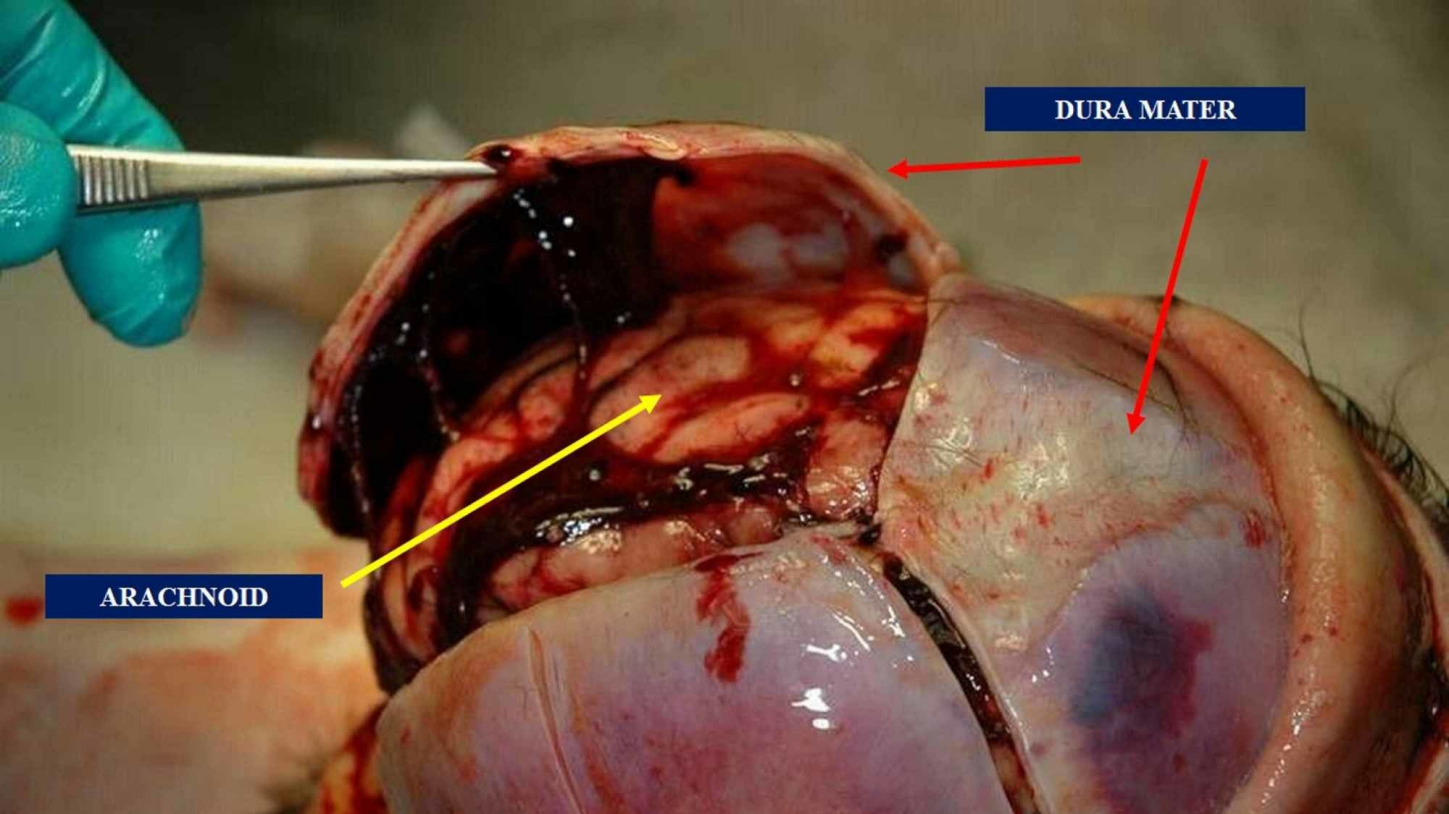
The image shows the dura mater and the superficial arachnoid layer. The outermost layer of the dura mater becomes continuous with the cranial periosteum presenting a strong adhesion to the base of the cranium and the foramen magnum, while the innermost layer communicates with the spinal dural system

Cranial dura mater, also known as the pachymeninx, is influenced by hormone level and changes its cellular constitution during metabolic fluctuations (animal model) (Figure 1) [8]. The outermost layer of the dura mater becomes continuous with the cranial periosteum presenting a strong adhesion to the base of the cranium and the foramen magnum, while the innermost layer communicates with the spinal dural system [9]. The internal dural folds originate reciprocal tension membranes: the falx cerebri; the tentorium cerebelli; the falx cerebelli; the diaphragm sellae [9]. The falx cerebri would tend to partially ossify, if not totally, with life progressing [4]. Literature has demonstrated that the falx cerebri is often shifted and directed toward one side (especially to the right), and it is not central. Literature reports cases of duplication and triplication of the falx cerebri [10]. The small falx or falx cerebelli might be absent or duplicated in pathological conditions [4]. The innervation of the dura mater is complex.

The innervation of the dura mater

The sympathetic innervation of the dura comes mainly from the postganglionic sympathetic fibers, ascending from the superior cervical ganglion, which follows the internal carotid and the meningeal arteries: the innervation concerns the area above it and the infratentorial region [1]. Animal studies have demonstrated a direct relationship between the dura and the stellate ganglion [1]. The supratentorial region, enclosed between the petrous part of the temporal bone and the diaphragma sellae, is innervated by the anterior and posterior branches of the ethmoidal nerves, arising from the maxillary and mandibular division of the trigeminal nerve [1]. The supratentorial region, enclosed between the petrous part of the temporal bone and the torcular herophili (internal occipital protuberance) is innervated by an ophthalmic branch or nervus tentorii [1]. The infratentorial regions are involved with the first two or three cervical branches, which enter through the foramen magnum and the hypoglossal canals, and directly through the hypoglossal and vagus cranial nerves. This area could cause local or referred pain if it was in dysfunction, whereas the pain would be distant from the original receptive field if the tentorial sinuses were involved in a disease process. The infratentorial region can cause heart arrhythmia and blood pressure issues for a cardio-trigeminal relation [11]. The dura mater is closely related to three out of the four suboccipital muscles (the two straight muscles, also known as recti, and the obliquus capitis superior muscle), the nuchal ligament and a spinal-nuchal ligament that relates the first cervical vertebra to the dura [12-13]. Some non-encapsulated receptors, with similar functions to the Ruffini endings, show a high response in both mechanical and chemical sensibility; they are located in the connective tissue of the dura mater, in proximity to the arterial and venous vessels [14]. These afferents also produce vasoactive substances, able to stimulate cerebral vasodilation [14]. The literature demonstrates that cicatricial tissues can be generated in the area between the vessels and connective tissue, stimulating further inflammation and vasodilatation; within the vessels, this can cause fibroblasts to turn into myofibroblasts, resulting in pain [8]. We know that some of the meningeal afferents concern extra-cranial tissues (periosteum and pericranial muscles) [14].

The arachnoid and pia mater layers

The arachnoid layer below the dura is thin and occupied by several trabecular structures; it has the function of shock absorber, towards the tensions received [15]. It is an avascular layer separated from the pia mater from the subarachnoid cavity filled with cerebrospinal fluid [16]. It surrounds blood vessels and nerves until they leave the cranial cavity, to become their epineurium. It follows the optic nerve all the way to the orbital cavity, where it merges with the sclera; it also has a close relationship with the olfactory nerve [16]. The arachnoid trabeculae contain strands of collagen tissues, which suggests a close relationship with nerve endings and this collagen because one of their variations in tension could determine afferents to be sent for cerebrospinal fluid regulation [16]. We have no exact information on the function of the pia mater [17]. It probably protects the cerebral substance and stabilizes the spinal cord, through denticulate ligaments, and it would have a role in the circulation of metabolites between the cerebrospinal fluid and surrounding structures [18]. The pia mater is located on the outermost layer of the brain, on a surface of glial cells, separated by a collagen-filled virtual space [18]. We know how fibroblasts are very malleable to mechanical stimulation [19]. A study on a corpse showed the falx cerebri shifting from a maximum of 1.44 mm (frontal lift) to a minimum of 0.28 mm (sphenobasilar decompression) [20]. Reciprocal tension membranes probably interact with the mechanical tensions arising from fluids, cerebral mass movements, and muscle structures.

Tentorium cerebelli

**Figure 2:**
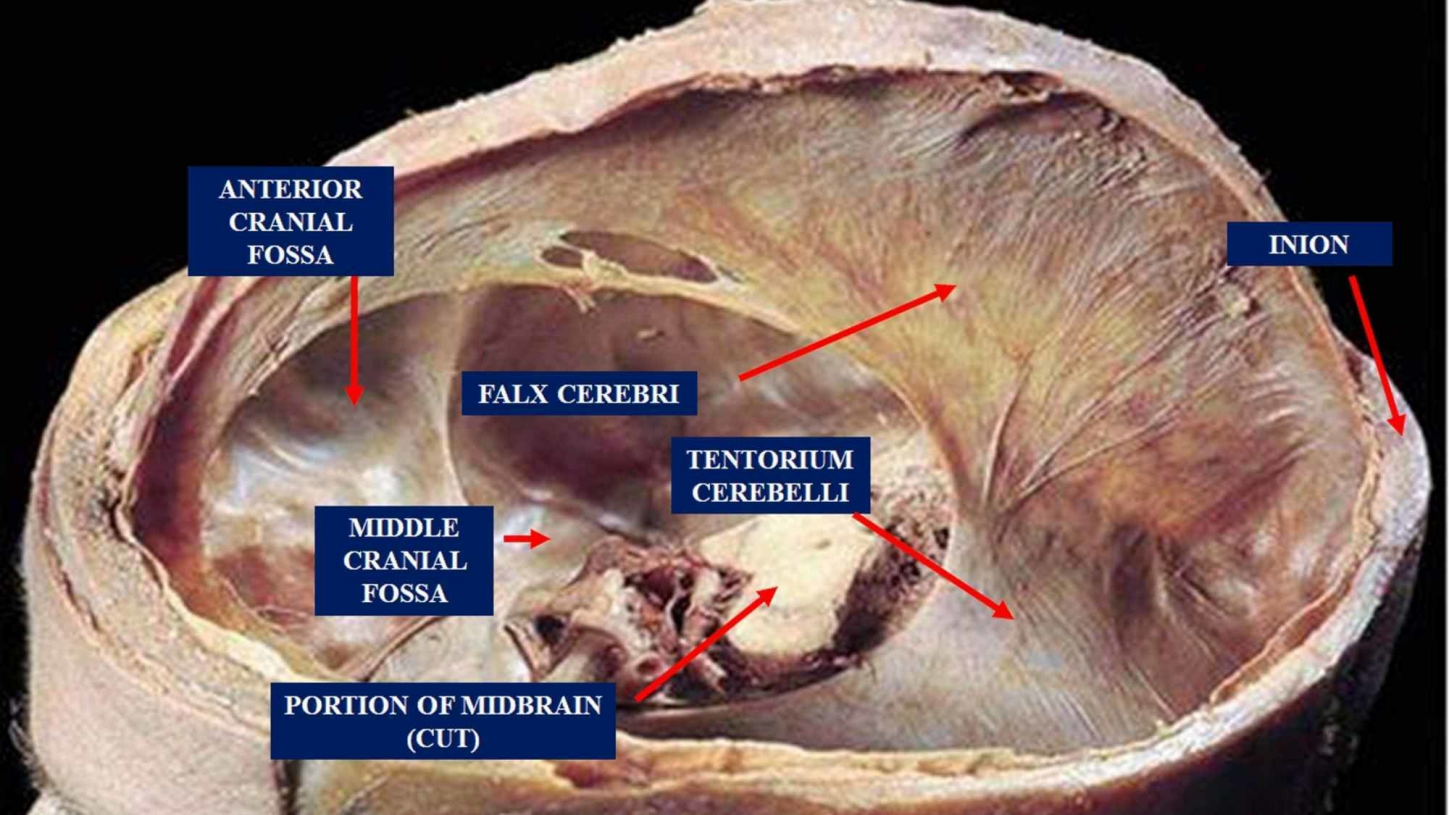
The figure shows the tentorium cerebelli, the falx cerebri and other portions of the skull. The tentorium cerebelli is located in the posterior cranial fossa; it is a semi-circular transverse septum covering the cerebellum, with occipital lobes of the cerebral hemispheres lying on it

The tentorium cerebelli is located in the posterior cranial fossa; it is a semi-circular transverse septum covering the cerebellum, with occipital lobes of the cerebral hemispheres lying on it (Figure 2) [21]. The falx cerebri is attached through its superior margin at the midline to the internal surface of the skull, while the falx cerebelli is below it. The anterior margin is concave, while the posterior margin is convex, which splits in half having an interior insertion on the superior margin of the petrous part of the temporal bone (containing the superior petrous sinus), while the posterior insertion is on the occipital squama and the parietal (containing the transverse sinus) [21]. The tentorium cerebelli delimits a specific area: from the internal occipital protuberance (where the sinus rectus is the continuation of the inferior sagittal sinus and is found along with the attachment of the falx cerebri to the tentorium cerebelli) to the temporal bone, constituting a semicircle [21]. The dural system contains a lymphatic system, called the glymphatic system. The cerebrospinal fluid is drained through the lymphatic and the venous systems [22-23]. Lymphatic vessels are present only in the dura mater, alongside the venous and arterial cerebral vessels; in particular, they pass through foramina of the skull following the reverse path of the pterygopalatine artery and a branch of the internal carotid, along the cranial external venous pathways and the cranial nerve routes [22]. The lymphatic vessels follow the venous pathways of the cribriform plate, to the nasal mucosa, through the cerebrospinal fluid exit pathways. The lymphatic vessels are mainly located at the base of the skull, compared to its apex. The glymphatic system absorbs the interstitial fluid and the cerebrospinal fluid from the subarachnoidal space, carrying it out from the base of the skull to the cervical tract; this mechanism is stronger during sleep [22]. The lymphatic system routes are subject to aging, losing its elasticity, and creating "aneurysms" over time, or decreasing the number of vessels or the lymphangions (the lymphatic functional unit) [23]. Recent evidence demonstrates that the lymphatic vessels are supported by a nervous system, vagal cholinergic and sympathetic, capable of modulating the contraction (peristalsis, aided by breathing and the pulsatility index of the arteries) of the vessels with contractile fibers (with an actin-like protein) [24]. These subtle nerves reach the outer layer of the lymphatic vessel, to finally innerve the deepest layer of endothelial cells; innervations decrements in elderly [24]. Probably, parasympathetic and sympathetic systems would function as sensors for vessel contractility and tension modulators or vascular tone of the vessel itself [24]. The sympathetic system, therefore, perceives the tension and is influenced by mechanical perception [25]. Patient’s body position influences cerebrospinal fluid and lymphatic drainage. Right side-lying body position facilitates cerebral lymphatic drainage (it increases vagal nerve modulation), while prone position would facilitate cerebrospinal fluid drainage (it increases sympathetic modulation) [26]. The dura mater is closely related to three out of the four suboccipital muscles (two recti capitis major and minor muscles, and the obliquus capitis superior and inferior muscles).

The tentorial bridge: muscles, ligaments, and dura mater

There are four sub-occipital muscles; three of these, the recti posterior major and minor, and the obliquus capitis inferior are connected to the dura mater by a myodural bridge in the proximity of the foramen magnum, through vertebral dural ligaments [27-31]. Such myodural bridge has proprioceptive receptors. The presence of proprioceptors is probably one of the causes of cervicocephalic headache and cervicocephalic pain syndromes [28,30]. The myodural bridge can transmit strong traction to the dural tissue during head movements [31]. The tension generated by the muscles and the dural area further helps the cerebrospinal fluid mobility [32]. A different structure connected to the dural tissue is the nuchal ligament (ligamentum nuchae) near the nuchal region [33]. This anatomic relationship affects head rotation on both sagittal and transverse planes [34]. Additional dural connections are known as Hoffman's ligaments, which are attached to the deep layer of the posterior longitudinal ligament [35]. Such relations, along with the entire dural sac, should allow adequate pressure for cerebrospinal fluid mobility, and avoid excessive stretching of the spinal nerves to prevent pain [35]. The yellow ligament is located behind the dural sac, starting from C1, showing strong dural connections [36]. One of its functional alterations will determine different cervical pathologies [37-38].

## Conclusions

The tentorium cerebelli is an integral part of the reciprocal tension membranes that divide some brain areas: the falx cerebri, the falx cerebelli, and the diaphragma sellae. The article discussed the anatomy of the tentorium cerebelli, the dura mater, and the ligaments and cervical muscles connected to the tentorium.
